# Biomechanical behaviour of tension-band-reconstruction titanium plate in open-door laminoplasty: a study based on finite element analysis

**DOI:** 10.1186/s12891-022-05804-w

**Published:** 2022-09-08

**Authors:** Hanpeng Xu, Jincheng Wu, Hongru Xie, Wangqiang Wen, Haoxiang Xu, Juan Du, Jun Miao

**Affiliations:** 1 Graduate School, Tianjin Medical University, Tianjin, China; 2 The First Affiliated Hospital of Hainan Medical University, Haikou, Hainan China; 3 The second people’s hospital of Hefei, Hefei, Anhui China; 4 Academy of Medical Engineering and Translational Medicine, Tianjin University, Tianjin, China; 5 Department of Spine Surgery, Tianjin Hospital, Jiefangnanlu 406, Hexi District, Tianjin, 300210 China

**Keywords:** Cervical spine, Open-door laminoplasty, Titanium plate, Finite element analysis

## Abstract

**Objective:**

To investigate and evaluate the biomechanical behaviour of tension-band-reconstruction (TBR) and ordinary titanium plates in open-door laminoplasty by finite element (FE) analysis.

**Methods:**

TBR titanium plate and ordinary titanium plate were implanted into a validated finite element model of healthy adult cervical vertebrae. Among them, 5 ordinary titanium plate were used in model A, 2 TBR titanium plates and 3 ordinary titanium plates were used in model B, and 5 TBR titanium plates were used in model C. The same loading conditions was applied identically to all models. Range of motion (ROM) of the vertebral body, stress distribution of the titanium plate and intradiscal pressure (IDP) were compared in flexion, extension, lateral bending and rotation.

**Results:**

The ROM of model B and C was similar in flexion and extension, and both were smaller than that of model A. The highest von Mises stress in the titanium plate appears is in model C. The IDP in C2/3 was significantly higher than that in other segments in flexion. There was no significant difference in IDP among three models in left lateral bending and left axial rotation.

**Conclusion:**

Application of TBR titanium plate in open-door laminoplasty can reduced ROM in flexion, extension and axial rotation of the cervical vertebrae. But the increase of stress in TBR titanium plate could lead to higher risk of adverse events such as titanium plate deformation. Moreover, compared with complete TBR titanium plate, the combination of TBR titanium plate for C3 and C7 with ordinary titanium plate for the other vertebrae largely reduce the stress of the titanium plates by ensuring stability. The proposed FE model (C2-T1) exhibits a great potential in evaluating biomechanical behaviour of TBR titanium plate for open-door laminoplasty.

**Supplementary Information:**

The online version contains supplementary material available at 10.1186/s12891-022-05804-w.

## Introduction

Cervical spondylotic myelopathy (CSM) is a degenerative spinal disease with clinical symptoms such as limb weakness and numbness [1]. Open-door laminoplasty is currently recognized as a classic surgical treatment for severe multisegment cervical spinal stenosis. In recent years, micro-titanium plate fixation has become widely recognized and used in Open-door laminoplasty [2]. Excellent biocompatibility, biomechanical performance, and most importantly simple operation procedure have been proofed by many surgeons [3].

In C3-C7 open-door laminoplasty, to fully expose the upper edge of the C3 lamina, the cervical semispinous muscle should be partially dissected at the C2 spinous process, and the spinous process of the operative segment together with the interspinous ligament (ISL) will be completely removed. The posterior cervical muscle-ligament complex (MLC) is an important structure for stability of the cervical vertebrae and mainly includes the spinous process, ISL and muscle tissue attached to the spinous process [4, 5]. When the MLC is seriously damaged, patients will experience symptoms of cervical weakness or even difficulty supporting the head in the short term; in the long term, this change is likely to lead to changes in the cervical curvature and atrophy of the posterior cervical muscles. Lin et al. [6] compared 53 patients with preservation of MLC and 37 patients with traditional open-door laminoplasty, and concluded that preserving MLC plays an important role in maintaining sagittal balance of cervical vertebra. Studies have shown that retaining one side of the MLC can reduce the incidence of axial symptoms (AS) [7]. Therefore, many studies continued to try to improve open-door laminoplasty.

Cheng et al. [8] removed the top of the C2 spinous process together with the attached muscles and then refixed the truncated tendon–bone structure to the spinous process after opening the lower lamina. This method provides greater strength than the method of fixing the muscle to the C2 spinous process with silk thread. Takeuchi et al. [9] applied C3 laminectomy combined with C4–7 open-door laminoplasty to eliminate the influence of the superimposed laminae without affecting the effect of spinal cord decompression. To preserve the C7 spinous process, Konig et al. [10] applied titanium plates for support and fixation on both sides of the lamina to reconstruct the integrity of the spinous process after laminectomy and decompression of the spinal cord and create conditions for muscle attachment.

Although retaining the C3 spinous process increases the attachment point, opening too few segments will limit the spinal cord decompression that can be achieved, and a new site of compression may easily form at the posterior and inferior border of the C3 lamina. Removal of the C3 lamina can avoid the dissection of muscle from the superior spinous process, but the integrity of the spinal canal is lost, and postoperative scar formation can lead to compression again. To the author’s knowledges, a simple operation method with decent postoperative effect is still under investigation.

**Fig. 1:**
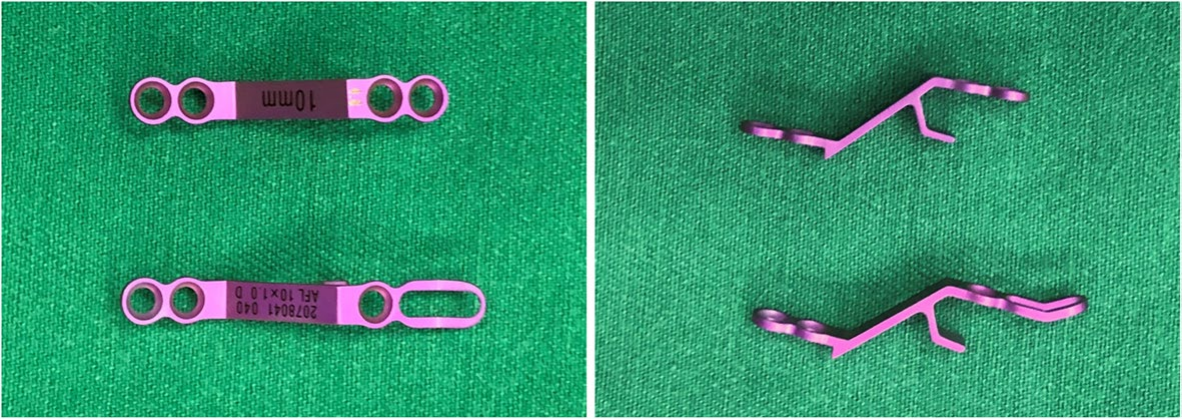
Front and side views of two titanium plates (lower, tension band titanium plate)

A tension-band-reconstruction (TBR) titanium plate for open-door laminoplasty was introduced for the first time (Fig. 1). The novel titanium plate has an innovative structure. The length of the titanium plate on the laminar side is appropriately extended, and an additional screw hole (three holes in total) is present at the distal end. During the operation, only the two inner holes are used for laminar fixation, while the remaining hole is used to suture the dissected muscle. This new type of titanium plate not only increases the stability of the door structure but also provides an attachment point for extensor reconstruction. From our previous clinical observation, the cross-sectional area of posterior cervical muscles was not significantly reduced after modified titanium microplate reconstruction, and the incidence of axial symptoms was 11.5%, which was lower than ordinary titanium plate (16.7%) [11]. However, the biomechanical behaviour of the new designed titanium plate, which can be the potential reason for this phenomenon is still unclear.

The aim of this study is to investigate the biomechanical behaviour of TBR titanium plate in open-door laminoplasty, generate reliable data for different spinal implants, and provide a potential tool to evaluate spinal implants.

## Materials and methods

### FE model of healthy adult cervical vertebrae (C2-T1)

In this study, a FE model of cervical spine (C2-T1) was build based on a healthy 26-year-old male volunteer. The absence of spinal lesions was confirmed on X-ray with no history of trauma or fracture. The volunteer was recruited from Tianjin Hospital in November 2020 and signed the volunteer’s informed consent form. The study was approved by the Ethics Committee of Tianjin Hospital. We used a 64-slice spiral CT scanner (Siemens, Erlangen, Germany) to obtain DICOM images of the C2-T1 vertebrae of the subject. The DICOM images were then imported into Mimics 20.0 (Materialise, Inc., Leuven, Belgium) to create a 3D vertebral surface model of C2-T1 and generate an STL file. Then, 3-matic 12.0 (Materialise, Inc.) was used to construct the posterior structures, intervertebral discs, nucleus pulposus and facet joints [12]. After these data were imported into the reverse engineering software Geomagic Studio 12.0 (Geomagic, Inc., USA) and materialized using smoothing, construction patches and grilles. Bones, discs and ligaments was meshed using Hypermesh2017 (Altair Engineering, Tr oy, Michigan, USA). Finally, the model was imported into Abaqus 2020 (Abaqus, Inc., USA) for FE analysis [13].

**Fig. 2:**
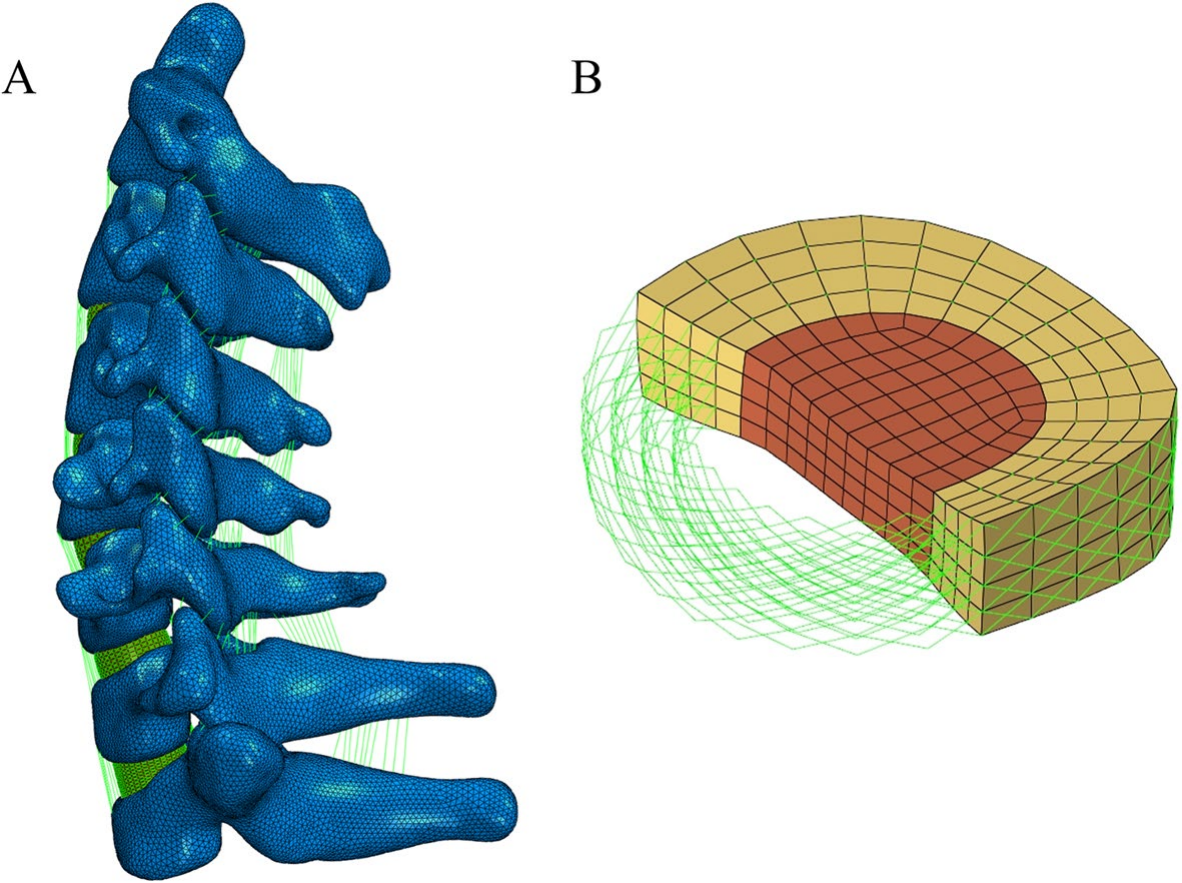
**A** Healthy adult cervical spine (C2-T1) FE model. **B** Intervertebral disc

Tetrahedral mesh was used on the vertebral body, and hexahedral mesh was used on the intervertebral disc (Fig. 2). The cortical bone, facet joint surface and cartilage endplate were meshed by shell elements, with thickness of 1.5 mm, 0.2 mm and 0.4 mm [14], respectively. The intervertebral disc was divided into the nucleus pulposus and the annulus fibrosus, with the nucleus pulposus accounting for 1/3 of the intervertebral volume [15]. The annulus fibrosus consists of an annulus fibrosus matrix and fibres. The inclination of the annular fibres relative to the horizontal plane is between 15° and 45°. The model simulated five ligaments: the anterior longitudinal ligament (ALL), posterior longitudinal ligament (PLL), ligamentum flavum (LF), interspinous ligament (ISL) and articular capsule ligament (CL). The annulus fibrosus and all intervertebral ligaments were modelled as tension-only truss elements. The contact between facet joints was designated as frictionless sliding [16]. The material properties used in the FE model (Table 1) were derived from the relevant literature [17–19].

**Table 1:** Material properties defined in the C2-T1 FE model

Component	Young’s modulus (MPa)	Poisson’s ratio	Cross-sectional area (mm^2^)
Bony structures			
Cortical bone	12,000	0.3	–
Cancellous bone	100	0.2	–
Endplate	500	0.4	–
Posterior structure	3500	0.25	–
Facet cartilage	10	0.4	–
Muscles	10	0.38	10
Intervertebral disc			
Annulus fibre	450	0.45	0.15
Annulus ground	Hyperelastic Mooney-Rivlin C10 = 0.56 C01 = 0.14		
Nucleus pulposus	Hyperelastic Mooney-Rivlin C10 = 0.12 C01 = 0.09		
Ligaments			
ALL	15	–	11.1
PLL	10	–	11.3
LF	5	–	46
ISL	4	–	12
CL	7	–	42.2
Implants			
Titanium plate/screw	110,000	0.3	–

### Model validation

The maximum von Mises stress of the model with element size of 1 mm, 1.5 mm and 2 mm is calculated and compared with the model with element size of 0.5 mm. When the difference is less than 5%, the element is considered to be convergent. In terms of the load and calculation accuracy, the element size of 1 mm is selected. In this case, the percentage error is 4.60%. The complete C2-T1 FE model had a total of 514,952 elements. Boundary conditions were applied to the lower surface of T1, and six degrees of freedom were fixed. The odontoid surface of C2 was coupled with the reference point at the upper 2 mm. A torque of 2.0 N·m was applied at this reference point to simulate flexion, extension, lateral bending and rotation of the cervical vertebrae. The range of motion (ROM) of each segment was measured and compared with previously reported results [20–23].

### C3-C7 laminectomy models

#### FE model of all ordinary titanium plates (model A)

**Fig. 3:**
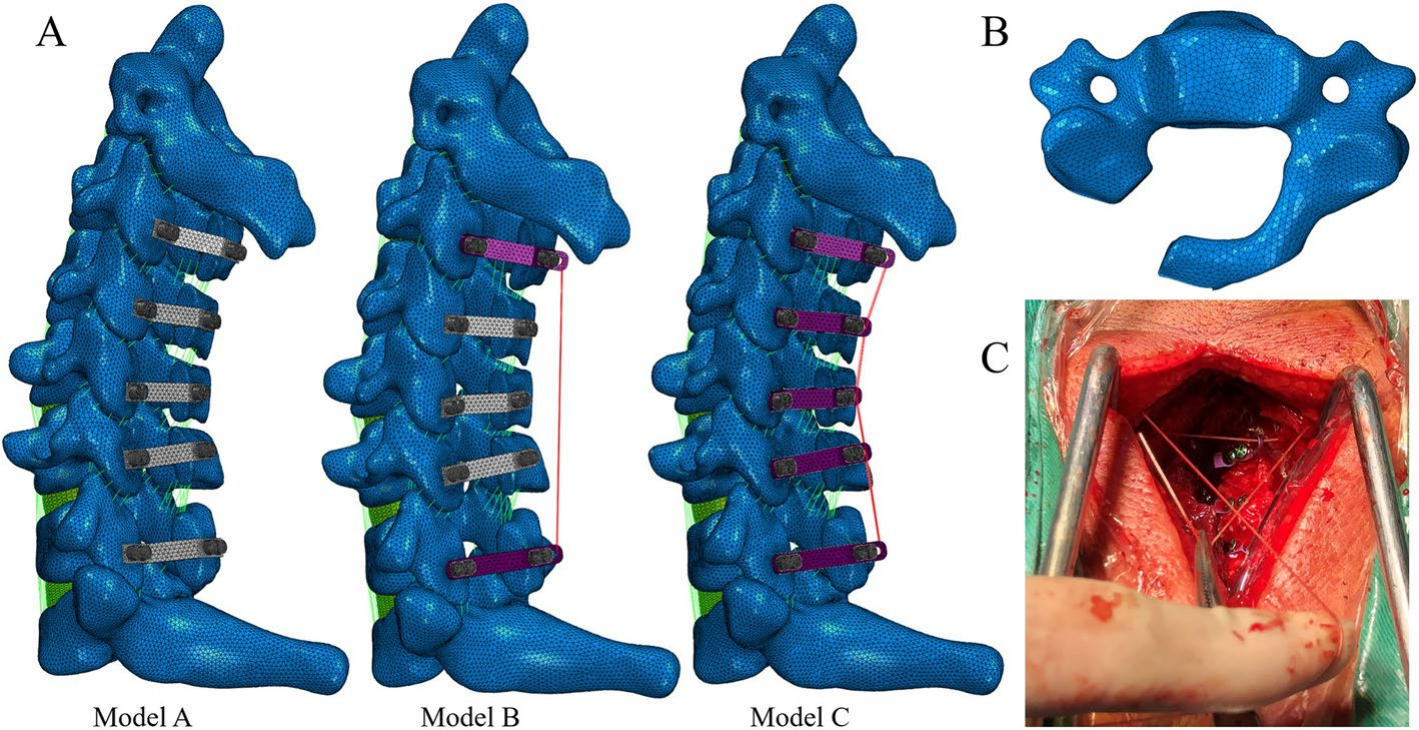
**A** FE model of open-door laminoplasty in three models. **B** Top view of C3 after open-door laminoplasty. **C** Intraoperative view of TBR titanium plate

In 3-matic, the spinous processes and laminae of C3-C7 of the verified normal adult cervical vertebral model were removed and opened, respectively. The operation to remove the spinous process is designed not to impede the degree of opening the door. After rematerialization, titanium plates and screws were implanted between the lateral mass and the lamina in Hypermesh; at the same time, the ISL and the LF were removed selectively. This completed the C2-T1 FE model of laminectomy (C3-C7) [7, 24], as shown in Fig. 3.

#### FE model of tension band titanium plate reconstruction for C3 and C7 (model B)

The TBR titanium plate based on the ordinary titanium plate features a long end loop; after the titanium plate was fixed behind the lamina and lateral mass with screws, the dissected cervical semispinalis muscle and multifidus muscle were sutured to the end loop of the C3 titanium plate, as shown in Fig. 3. During the operation, the cranial and caudal levels of the open-door segment can be reconstructed with a TBR titanium plate to achieve the effect of MLC reconstruction and increase stability of the cervical spine after the operation.

Using the FE model mentioned in the previous section, the ordinary titanium plates at C3 and C7 were replaced by TBR titanium plates. These plates were fixed with screws, and the end loops of the two titanium plates were connected by T3D4 elements and assigned muscle properties. This completed the FE model of laminectomy with partial TBR titanium plate [25], as shown in Fig. 3.

#### FE model of all tension band titanium plates (model C)

In open-door laminoplasty, TBR titanium plates can also be used at each level of the operative segment. After the titanium plate was fixed, the dissected cervical semispinalis muscle and multifid muscle were sutured to the end loop of the C3 titanium plate, and the rest of the posterior cervical muscle group was sutured to the end loop of the other titanium plate.

All the ordinary titanium plates in the FE model above were replaced with TBR titanium plates, as shown in Fig. 3, which were connected one by one with a T3D4 element and assigned muscle properties. After the screws and titanium plates were attached to the vertebral bodies, the FE model of open-door laminoplasty with five TBR titanium plates was completed.

#### Biomechanical performance analysis

We applied the same load to the above three models and used a moment of 2.0 N·m to simulate the flexion, extension, lateral bending and axial rotation of cervical vertebrae. According to the simulation in ABAQUS, the ROM of each cervical segment and the stress distribution contour of each titanium plate were recorded. Because only one subject was modelled, there was no statistical analysis in this study.

## Results

### Validation of the healthy adult C2-T1 FE model

**Fig. 4:**
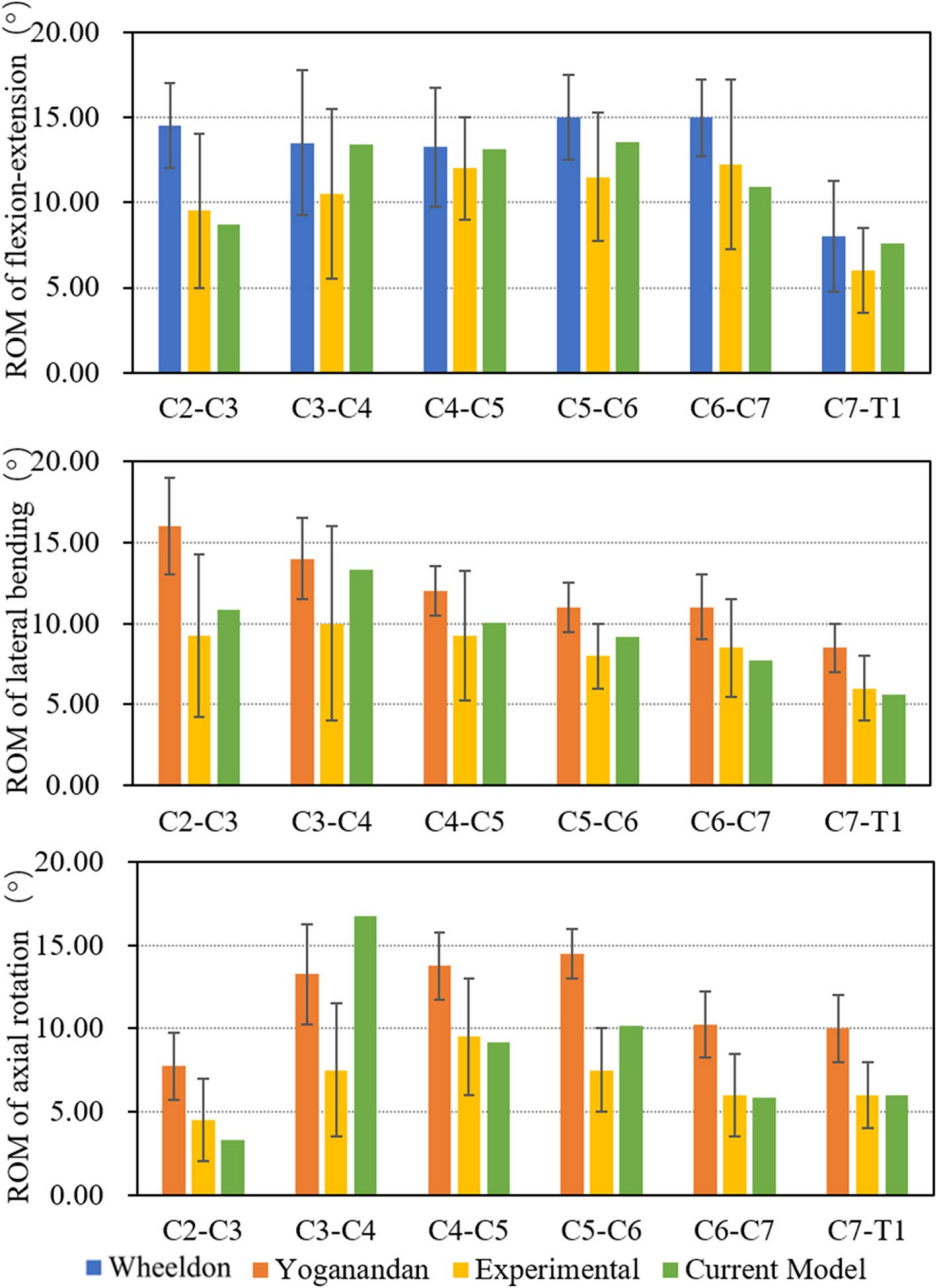
Comparison of level-by-level ROM for intact FE model between in-house experimental and reported data in flexion/extension, lateral bending and axial rotation at 2 N·m

ROM was calculated for each cervical segment of the FE model in six directions in flexion, extension, lateral bending and rotation with a 2.0 N·m moment (Fig. 4). Compared with previous in vitro experiments and FE studies, ROM obtained from our model were in the same range of those reported by Wheeldon et al. [22], Yoganandan et al. [26] for all flexion, extension and lateral bending. In rotation, the intact model predicted motions that were comparable to the experimental data for the majority of loading modes [18, 21, 27, 28]. Thus, a reliable healthy adult C2-T1 FE model was obtained in accordance with human physiological conditions .

### Biomechanical behaviour of TBR titanium plate

#### ROM of the cervical spine

**Fig. 5:**
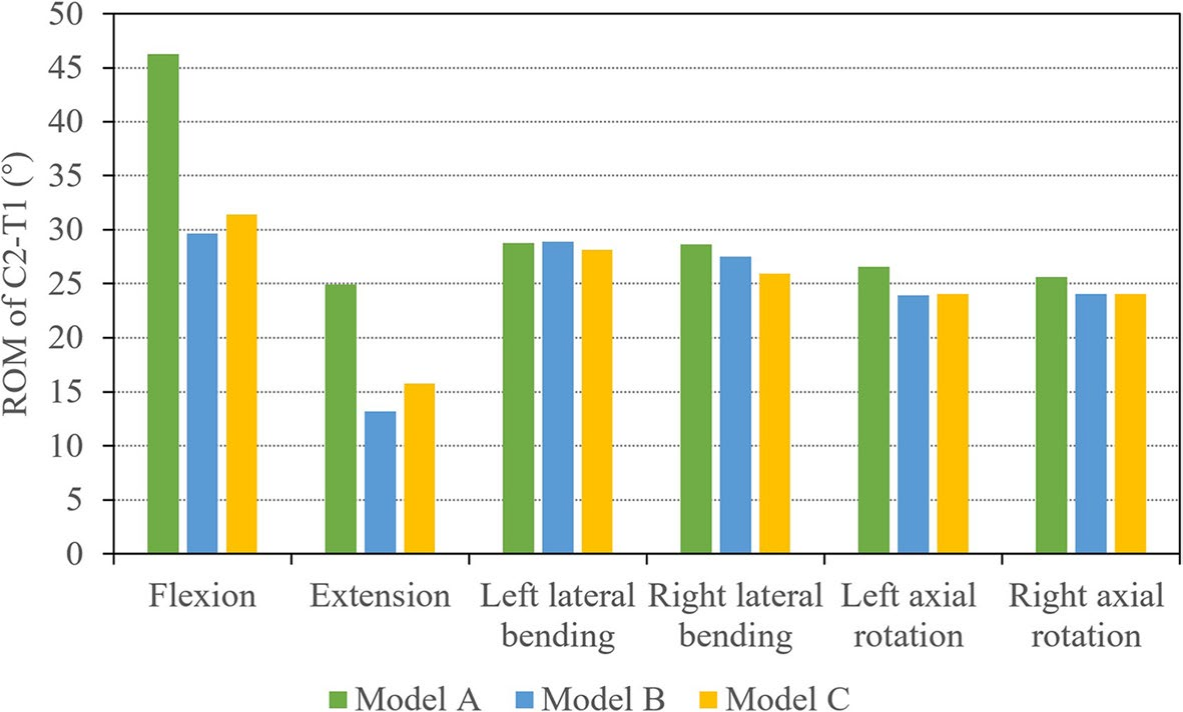
ROM of C2-T1 (°) in the three models in flexion, extension, lateral bending and axial rotation

At the same moment, the overall ROM in model B and C with TBR titanium plate in flexion was smaller than that in model A, and the ROM in model B was the lowest for all three types of models. (Fig. 5). The ROM in model B in flexion was 35.97% lower than that in model A, while that in model C was 31.76% lower than that in model A. Similarly, three models showed the same trend in extension. The ROM in model B was 46.99% lower than that in model A, while the ROM in model C was 36.77% lower than that in model A. The ROM of the three models were 28.61°, 27.5°, and 25.92° in right lateral bending. However, both model B and C were smaller than model A in axial rotation. There was little difference in axial rotation between model B and model C.

#### Stress distribution of the titanium plates

**Fig. 6:**
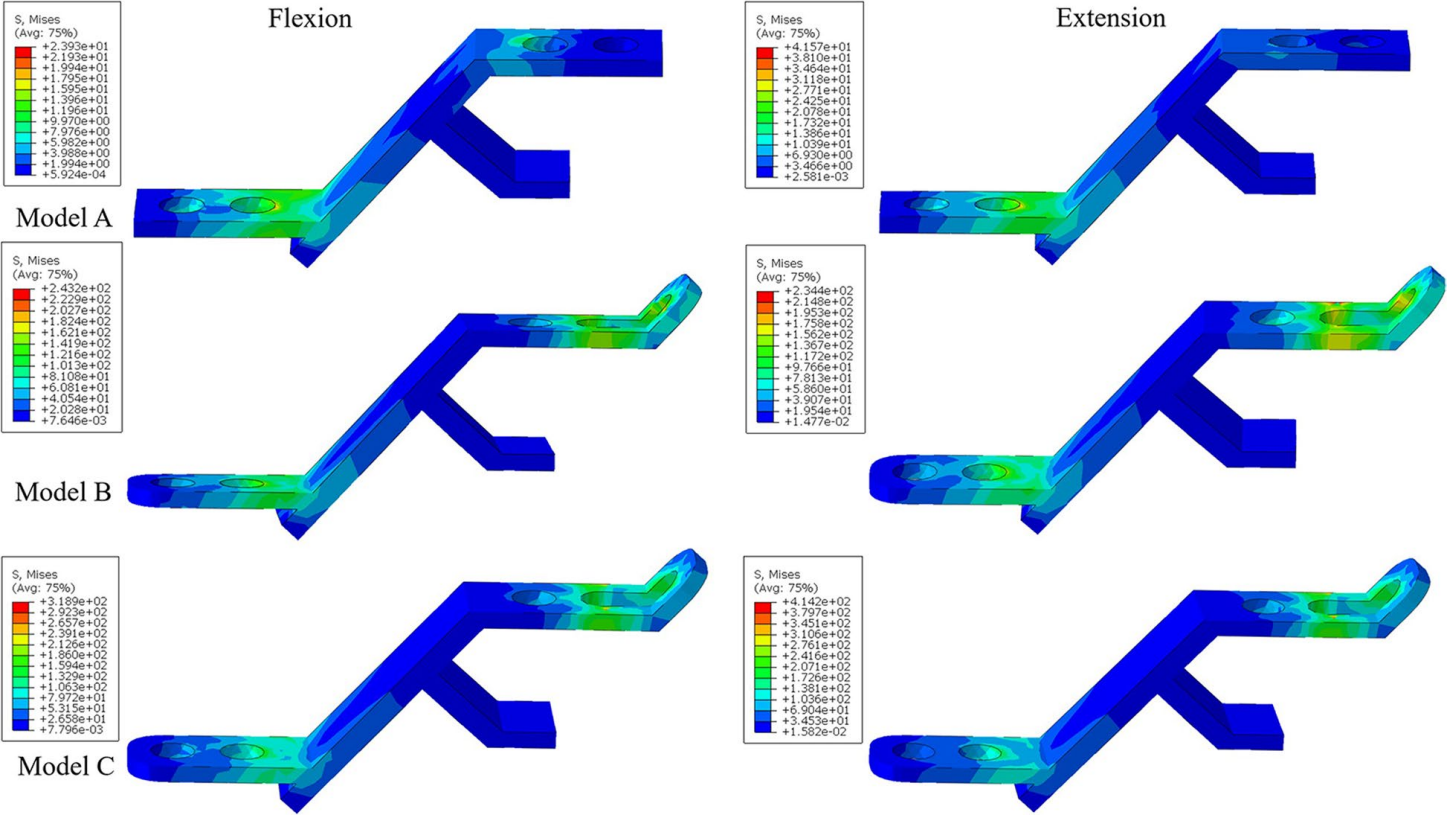
Von-Mises stress (MPa) distribution in titanium plates at C3 in flexion and extension in the three models

**Fig. 7:**
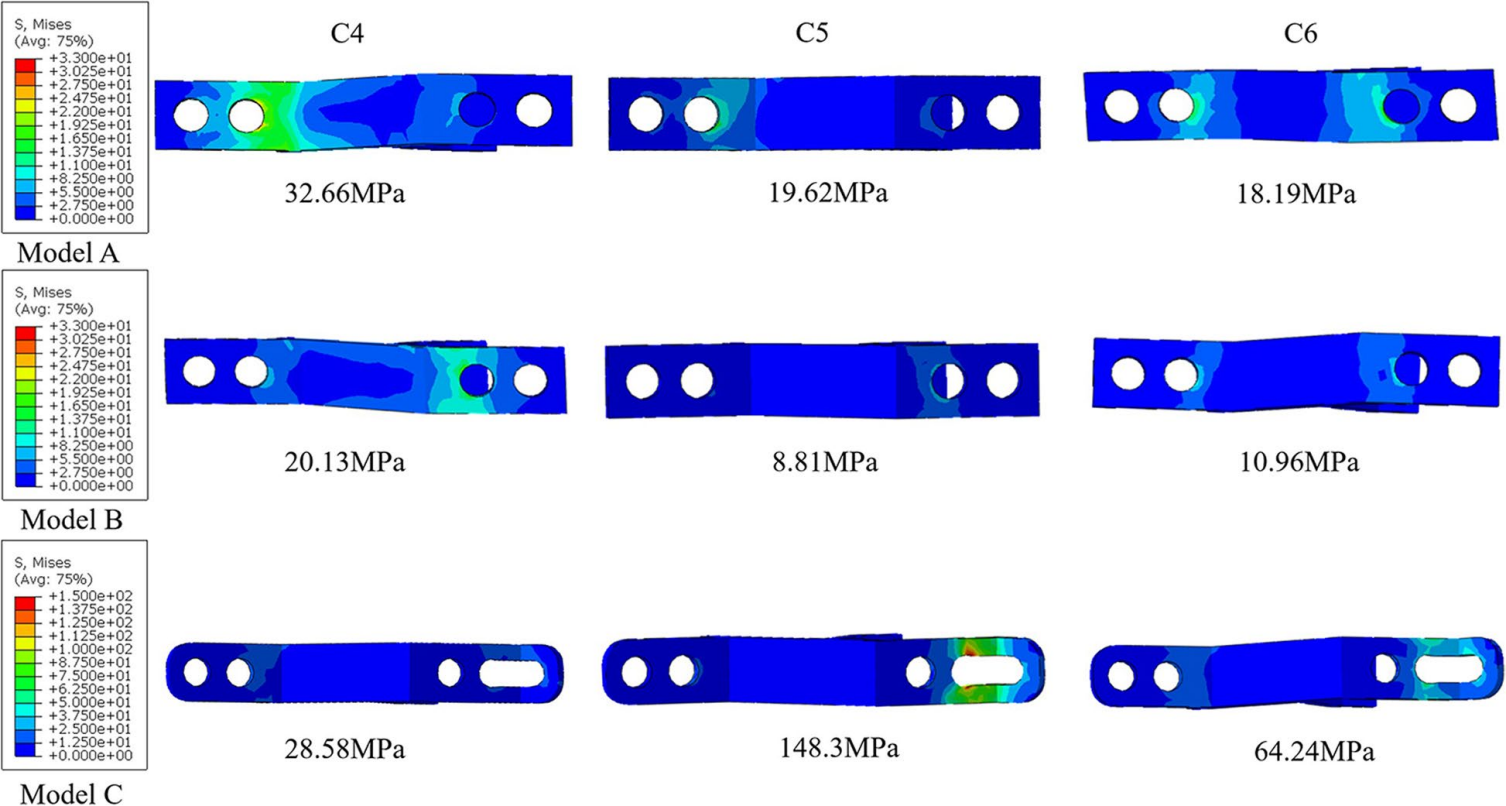
Von-Mises stress (MPa) distribution and the value of maximum stress in titanium plates at C4, C5 and C6 in three models in flexion

The maximum stress in the titanium plates in the three models in each motion showed an increasing trend. Among them, the greatest increase in stress was observed in flexion and extension (Fig. 6). In flexion, the stress in model B reached 259.1 MPa, while that in model C reached 318.9 MPa. In extension, the stress in model B reached 320.7 MPa, while that in model C reached 414.2 MPa. In flexion and extension in model B, the stress of titanium plates located at C4, C5 and C6 was lower than that of plates at C3 and C7 (Fig. 7). In lateral bending, the maximum stress of the titanium plates in model C was 34% higher than that in model B, while it was 26% higher in axial rotation. The maximum von-Mises stress of each titanium plate during flexion, extension, lateral bending and rotation was presented in the Additional file 1: Appendix 1.

#### Intradiscal pressure

**Fig. 8:**
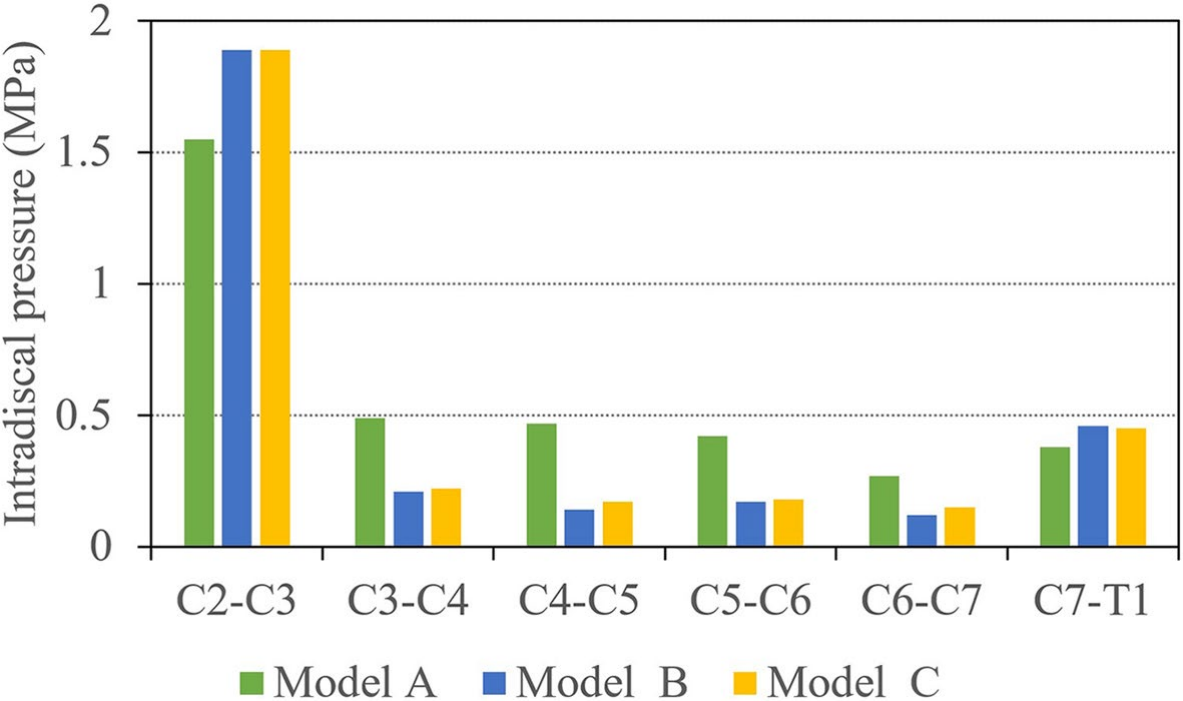
Intradiscal pressure (MPa) in the three models in flexion

The intradiscal pressure (IDP) at C2/3 of model A, B and C were 1.55 MPa, 1.89 MPa, and 1.89 MPa in flexion (Fig. 8). While the IDPs of model B and C were smaller than that of model A at C3/4, C4/5, C5/6 and C6/7. The three models showed the same trend in extension. The corresponding IDPs at C2/3 were 0.37 MPa, 0.46 MPa, and 0.46 MPa. There is little difference among the three models in left lateral bending and left axial rotation. The IDPs of model B and C at C2/3 were higher than that of model A in right lateral bending and right axial rotation. The IDPs of model A, B and C at C2/3 in right lateral bending were 0.43 MPa,0.84 MPa and 0.83 MPa, respectively. The IDPs of model A, B and C at C2/3 in right axial rotation were 0.46 MPa,0.76 MPa and 0.73 MPa, respectively. The IDPs of three models during flexion, extension, lateral bending and rotation were presented in the Additional file 1: Appendix 2.

## Discussion

In this study, FE models of three different combinations of titanium plates in cervical spine open-door laminoplasty were built to evaluate their biomechanical behaviour. We found TBR titanium plates yielded better stability in flexion, extension and axial rotation. Comparing with model A, the ROM of model B in flexion and extension was 35.97 and 46.99% lower; while 31.76 and 36.77% lower in model C than in model A, respectively. At the same time, the stress of the titanium plates was largely increased when too many TBR titanium plates were used. The IDP and titanium plate stress showed the same trend. The IDP of C2/3 and C7/T1 in model B and C in flexion and extension were slightly higher than that in model A, while IDP in the middle segment decreased. Thus, in model B which used a combination of TBR titanium plates for C3 and C7 and ordinary titanium plates for the other vertebrae, showing less burden on the titanium plates, but ensure good stability and reduced the stress of the titanium plates and IDPs in the middle of the segment.

It has been widely approved that the semispinalis cervicalis is the most important extensor muscle in the posterior cervical muscles. Its proximal end mainly stops at the C2 spinous process, and the posterior extension force generated during contraction accounts for 37% of the total contraction force of the posterior cervical muscles [29]. Therefore, the mechanical stabilization device composed of cervical semispinalis muscle and C2 spinous process plays an extremely important role in maintaining the posterior tension and physiological kyphosis of the cervical spine. The results of FEA show that due to the fact that the TBR titanium plate provides an extra hole to reconstruct the muscle attachment point, the muscle separated during the operation is re-attached to C3. This increases the stability of the cervical spine in the direction of flexion, extension and rotation analysis after open-door operation. Because the motion plane of the cervical vertebrae during lateral bending is not consistent with the direction of the reconstructed muscles, the use of TBR titanium plate has little effect on the ROM of lateral bending. The inconsistency in the ROM of three models during right lateral bending may be related to the opening angle of the lamina.

The stress distribution of titanium plate in three models shows substantial different pattens. As the new titanium plate provides muscle attachment points, the titanium plate stress rises. In model B, the stress of the titanium plates in the middle of the segment was partially transferred to the cranial and caudal ends of the segment. However, model C applied five TBR titanium plates, and the stress of the titanium plate increased significantly, which may be related to the increase of the force arm. It is worth noting that the new titanium plate has stress concentration near the newly added hole, which may lead to a negative effect on the durability of the titanium plate. Therefore, the fatigue test of the new titanium plate needs to be further studied.

The IDP of C2–3 increased significantly in the three models during flexion. This may be due to the removal of the LF in C2–3 and most of the ISL, and the lack of posterior structural stress during flexion, resulting in stress concentration in the anterior column of the vertebral body. This suggests a clinical risk of disease at this level. This study, however, simulated only a portion of the muscle, which may have magnified the phenomenon. In lateral bending and axial rotation, the inconsistency between left and right of IDP may be related to the opening direction of open-door laminoplasty.

It is particularly important to preserve or reconstruct the muscle attachment point and ligament complex in posterior cervical open-door laminoplasty [30–32]. However, every attempt to retain or reconstruct the attachment point and ligament complex may potentially increase the difficulty of opening the lamina and the complexity of the surgical operation. TBR titanium plate adopts a simple method to achieve MLC reconstruction without increasing the operative duration or intraoperative blood loss.

This study compared the biomechanical performance of different plates, which provide a non-invasively tool to evaluate their mechanical performance during human movement. However, there are still some limitations: first, follow-up promotion is still needed to be confirmed by a multicentre controlled large-sample size. In addition, we didn’t take muscle into account during validation. However, the three models are not compared with the complete model when they are compared with each other. Model B and model C used TBR titanium plate and simulated muscle, while model A did not. The comparison of the three models can show the difference between TBR titanium plate and ordinary titanium plate. And there is a lack of corroborative research in vitro to improve the simulation model and establishing finer muscle simulations. In our future research plan, in vitro biomechanical experiments will also be included. At last, the model created in this study shown a great potential in evaluating mechanical performance of different types of fixators.

## Conclusion

In this study, a healthy adult cervical spine (C2-T1) FE model and corresponding open-door laminoplasty (C3-C7) FE models were established and used for further biomechanical analysis. FE analysis showed better stability using TBR titanium plate in open-door laminoplasty. But the increase of stress in TBR titanium plate could lead to a higher risk of adverse events such as titanium plate deformation. Compared with complete TBR titanium plate, the combination of TBR titanium plates for C3 and C7 with ordinary titanium plates for the other vertebrae largely reduce the stress of the titanium plates by ensuring stability.

## Supplementary Information

**Additional file 1: Appendix1.** The maximum von stress (MPa) of the titanium plate on each segment. **Appendix2.** The intradiscal pressure (MPa) of three groups.

## Data Availability

The datasets generated and/or analyzed during the current study are not publicly available due the requirements of patent protection but are available from the corresponding author on reasonable request.

## Abbreviations

FE: Finite element
TBR titanium plate: Tension-band- reconstruction titanium plate
ROM: Ranges of motion
IDP: Intradiscal pressure
MLC: Muscle-ligament complex
ISL: Interspinous ligament
