# Supplementary material for: Biomechanical behaviour of tension-band-reconstruction titanium plate in open-door laminoplasty: a study based on finite element analysis
Source: BMC Musculoskelet Disord. 2022 Sep 8;23:851. doi: 10.1186/s12891-022-05804-w (PMC9454233; doi:10.1186/s12891-022-05804-w)
Supplement: Supplementary file 1 — Additional file 1: Appendix1. The maximum von stress (MPa) of the titanium plate on each segment. Appendix2. The intradiscal pressure (MPa) of three groups. [file 12891_2022_5804_MOESM1_ESM.docx]

|  |  | Flexion | Extension | Left lateral bending | Right lateral bending | Left axial rotation | Right axial rotation |
| --- | --- | --- | --- | --- | --- | --- | --- |
| C3 | Group A | 23.93 | 41.57 | 11.94 | 23.67 | 95.31 | 125.7 |
|  | Group B | 244.5 | 320.7 | 24.5 | 87.13 | 131.2 | 84.91 |
|  | Group C | 318.9 | 414.2 | 33.04 | 138.6 | 105.5 | 62.06 |
| C4 | Group A | 32.66 | 31.44 | 13.95 | 19.43 | 98.35 | 98.11 |
|  | Group B | 20.13 | 5.71 | 33.89 | 34.13 | 73.48 | 131.2 |
|  | Group C | 28.58 | 35.24 | 23.56 | 19.68 | 53.14 | 108.1 |
| C5 | Group A | 19.62 | 75.05 | 89.7 | 31.86 | 69.85 | 70.83 |
|  | Group B | 8.81 | 7.18 | 116.2 | 43.71 | 102.5 | 85.54 |
|  | Group C | 148.3 | 261.9 | 87.56 | 57.62 | 53.51 | 38.85 |
| C6 | Group A | 18.19 | 18.21 | 58.37 | 43.73 | 96.94 | 111 |
|  | Group B | 10.96 | 11.8 | 84.09 | 95.52 | 106 | 130.1 |
|  | Group C | 64.24 | 67.61 | 41.23 | 71.62 | 44.36 | 72.96 |
| C7 | Group A | 29.95 | 8.026 | 59.85 | 62.5 | 57.96 | 55.21 |
|  | Group B | 259.1 | 309.3 | 47.91 | 67.16 | 46.29 | 56.5 |
|  | Group C | 158.6 | 190 | 50.08 | 34.24 | 25.55 | 63.75 |

Appendix1. The maximum von stress (MPa) of the titanium plate on each segment

Appendix2. The intradiscal pressure (MPa) of three groups

|  |  | Flexion | Extension | Left lateral bending | Right lateral bending | Left axial rotation | Right axial rotation |
| --- | --- | --- | --- | --- | --- | --- | --- |
| C2-C3 | Group A | 1.55 | 0.37 | 0.67 | 0.43 | 0.37 | 0.46 |
|  | Group B | 1.89 | 0.46 | 0.56 | 0.84 | 0.31 | 0.76 |
|  | Group C | 1.89 | 0.46 | 0.56 | 0.83 | 0.32 | 0.73 |
| C3-C4 | Group A | 0.49 | 0.29 | 0.27 | 0.13 | 0.22 | 0.26 |
|  | Group B | 0.21 | 0.16 | 0.3 | 0.33 | 0.16 | 0.14 |
|  | Group C | 0.22 | 0.15 | 0.25 | 0.23 | 0.12 | 0.09 |
| C4-C5 | Group A | 0.47 | 0.41 | 0.34 | 0.43 | 0.41 | 0.38 |
|  | Group B | 0.14 | 0.14 | 0.34 | 0.39 | 0.33 | 0.33 |
|  | Group C | 0.17 | 0.22 | 0.35 | 0.35 | 0.32 | 0.37 |
| C5-C6 | Group A | 0.42 | 0.24 | 0.15 | 0.25 | 0.22 | 0.16 |
|  | Group B | 0.17 | 0.11 | 0.15 | 0.14 | 0.18 | 0.24 |
|  | Group C | 0.18 | 0.14 | 0.17 | 0.17 | 0.21 | 0.23 |
| C6-C7 | Group A | 0.27 | 0.26 | 0.24 | 0.48 | 0.28 | 0.34 |
|  | Group B | 0.12 | 0.1 | 0.23 | 0.39 | 0.25 | 0.43 |
|  | Group C | 0.15 | 0.14 | 0.23 | 0.35 | 0.26 | 0.34 |
| C7-T1 | Group A | 0.38 | 0.15 | 0.17 | 0.31 | 0.28 | 0.18 |
|  | Group B | 0.46 | 0.16 | 0.22 | 0.2 | 0.36 | 0.33 |
|  | Group C | 0.45 | 0.16 | 0.22 | 0.2 | 0.34 | 0.34 |
